# Association of sleep efficiency with cardiac autonomic modulation in young and professional soccer players during pre-season

**DOI:** 10.1007/s11325-026-03811-y

**Published:** 2026-09-18

**Authors:** Nathaniel Gomes Oliveira, Lucas Miquéias Silva Abreu, Felipe Bispo Ribeiro, Bianca Fernanda Almeida Silva, Tanara Nubia Furtado Monteiro, Michele Brito Correia, Manuelly Estefanny Vieira Pereira, Herickson Araújo Costa, Cristiano Teixeira Mostarda, Carlos Alberto Alves Dias Filho, Jefferson Fernando Coelho Rodrigues, Janaina de Oliveira Brito Monzani, Emerson Silami Garcia, Christian Emmanuel Torres Cabido, Carlos José Moraes Dias

**Affiliations:** 1 Postgraduate Program in Physical Education, Centro de Ciências da Saúde, Federal University of Maranhão, Avenida dos Portugueses, Vila Bacanga, São Luís, MA 65080805 Brazil; 2 Department of Physical Education, Federal University of Maranhão (UFMA), Pinheiro, MA Brazil; 3 Department of Physical Education, Federal University of Maranhão (UFMA), São Luís, MA Brazil; 4 Postgraduate Program in Health Biotechnology (RENORBIO), Federal University of Maranhão, São Luís, MA Brazil; 5 Postgraduate Program in Adult Health (PPGSAD/UFMA), Federal University of Maranhão, São Luís, MA Brazil; 6 Postgraduate Program in Health Sciences (PPGCS/UFMA), Federal University of Maranhão, São Luís, MA Brazil; 7 Laboratory of the Physical Exercise Research Group, Health and Human Performance, São Luís, MA Brazil; 8 Laboratory of Cardiorenal Adaptations to Physical Exercise, Pinheiro, MA Brazil

**Keywords:** Heart rate variability, Sleep quality, Soccer, Pre-season, Autonomic nervous system

## Abstract

**Objective:**

This study investigated the association between sleep efficiency and cardiac autonomic modulation in young and professional soccer players during pre-season.

**Methods:**

A cross-sectional quantitative study was conducted with 53 male soccer players divided into three categories: U-15 (*n* = 12), U-17 (*n* = 15), and professionals (*n* = 26). Sleep quality was assessed using the Pittsburgh Sleep Quality Index (PSQI), cardiac autonomic modulation via heart rate variability (HRV) recorded by a 10-lead electrocardiograph and analyzed with Kubios HRV 2.0, and cardiorespiratory capacity estimated through the Yo-Yo Intermittent Recovery Test. Body composition was assessed by tetrapolar electrical bioimpedance. Statistical analyses included one-way ANOVA, Welch’s ANOVA, chi-square and Fisher’s exact tests, Pearson correlation, and simple linear regression, with significance set at *p* < 0.05.

**Results:**

Professional athletes demonstrated significantly higher VO₂max (50.5 ± 4.56 ml·kg⁻¹·min⁻¹; *p* < 0.001), lower resting heart rate (54.9 ± 8.65 bpm; *p* = 0.03), and greater parasympathetic modulation (RMSSD: 82.8 ± 17.5 ms; *p* < 0.001) compared to youth categories. Sleep efficiency was significantly higher in professionals (90.7 ± 6.13%; *p* = 0.01) and positively correlated with RMSSD. A significant association was found between RMSSD levels and global sleep quality (*p* = 0.004; X² = 0.50), as well as between RMSSD and VO₂max classification (*p* = 0.01; X² = 0.40).

**Conclusion:**

Sleep efficiency is positively associated with cardiac autonomic modulation and aerobic performance in soccer players. While the superior physiological profile of professionals reflects a synergy between training adaptations and biological maturation, HRV and PSQI constitute complementary monitoring tools for integrated athlete assessment across competitive categories.

## Introduction

The physical performance of elite athletes is determined by complex physiological interactions, in which sleep quality, autonomic regulation, and aerobic conditioning play a central role in modulating responses to intense exercise and training adaptation [1]. Among training periods, the pre-season represents a critical phase for metabolic and physical adaptations, changes in sympathovagal balance, and sleep architecture, factors that significantly impact recovery capacity and athletic performance [2, 3]. In this context, sleep quality influences energy homeostasis and tissue repair, and the Pittsburgh Sleep Quality Index (PSQI) is recognized as a relevant indicator of athletic performance, good recovery, and injury risk reduction [4].

Competitive athletes with compromised sleep quality (PSQI > 5) experience negative impacts on essential physiological functions and a higher incidence of injuries [5]. Young athletes, in particular, due to accelerated maturation of the autonomic nervous system (ANS), are more susceptible to sleep deprivation, presenting reduced vagal activity and altered autonomic modulation [6]. Furthermore, inadequate sleep patterns are inversely related to athletic performance and act as a critical predictor for musculoskeletal injuries [7]. Poor sleep quality is also associated with reduced maximum oxygen consumption (VO2max) [8], and chronic sleep deprivation triggers a systemic pro-inflammatory state that impairs endothelial function and tissue perfusion [9, 10].

Cardiac autonomic regulation assessed via heart rate variability (HRV) serves as a sensitive marker of physiological stress in athletes [11], influenced by chronic training effects and cardiovascular efficiency [12]. Inadequate sleep disrupts this modulation, intensifying sympathetic activity and reducing parasympathetic tone, leading to increased resting heart rate and decreased HRV, which may indicate cardiovascular risk [13, 14].

Although recent literature recognizes the impact of sleep quality on cardiac autonomic modulation in athletes [15, 16], a gap remains in direct comparisons between young and professional soccer players during the pre-season. It remains unclear how differences in ANS maturation between categories modulate physiological responses to sleep restriction. Thus, this study aims to investigate the association between sleep efficiency and cardiac autonomic modulation in young and professional soccer players during pre-season.

## Methods

### Study design and ethics

This study adopted a cross-sectional quantitative design. Data collection was conducted in four sequential phases: (1) anamnesis and application of validated questionnaires (IPAQ and PSQI); (2) body composition assessment by electrical bioimpedance and resting electrocardiogram; (3) cardiorespiratory capacity assessment via the Yo-Yo Intermittent Recovery Test; and (4) data analysis and individual feedback to participants.

### Participants

Fifty-three of male soccer players were recruited by convenience sampling [17] from two institutions in Pinheiro, MA, divided into three groups: U-15 (*n* = 12; 14–15 years), U-17 (*n* = 15; 16–17 years), and professionals (*n* = 26; 18–28 years). All participants were in their pre-season period and voluntarily agreed to participate following established ethical procedures. Data collection was conducted during the second week of the pre-season. Professional status was defined as athletes aged ≥ 18 years with a formal professional contract and active participation in regional-level competitions. The professional team’s pre-season lasted 4 weeks, comprising a formally structured program supervised by certified coaching and sports science staff, with periodized aerobic, strength, and tactical training sessions. In contrast, the U-15 and U-17 categories underwent an approximately 3-week preparatory period, composed predominantly of technical-tactical sessions with less structured periodization and lower training load monitoring, consistent with their developmental stage and institutional resources.

### Inclusion criteria

- Male soccer players were actively engaged in pre-season training at the time of data collection.
- Registered in one of the three competitive categories: U-15, U-17, or professional.
- Medically cleared for physical activity, with no contraindications identified during anamnesis.
- Able to complete all data collection stages in full.
- Voluntary agreement to participate, formalized by signing the Informed Consent Form (ICF); for participants under 18 years, consent was also obtained from parents or legal guardians.

### Exclusion criteria

- Use of medications or supplements known to affect autonomic function or cardiovascular parameters.
- Presence of musculoskeletal injuries or clinical conditions preventing maximal effort testing.
- History of diagnosed sleep disorders (e.g., insomnia, sleep apnea).
- Failure to complete any of the four data collection phases.
- Voluntary withdrawal from the study at any stage.

### Physical activity level

The short form of the International Physical Activity Questionnaire (IPAQ) was applied, as validated for Brazilian populations [18, 19].

### Sleep quality

The Pittsburgh Sleep Quality Index (PSQI) was used to assess sleep quality across seven components: subjective quality, latency, duration, habitual efficiency, disturbances, medication use, and daytime dysfunction, yielding a global score from 0 to 21 points. Scores above 5 indicate poor sleep quality [20] Although direct sleep measurements (e.g., polysomnography or actigraphy) are considered gold standards, the PSQI was chosen as a practical, cost-effective, and non-invasive indirect measure that does not disrupt the athletes’ recovery routines. We administered the validated [Portuguese] version of the PSQI, as all participants were native speakers and fully familiar with the language. This version has demonstrated adequate reliability and validity for assessing sleep in athletic populations [21]. The use of non-prescription sleep aids or specific sleep medications was recorded but did not constitute an exclusion criterion, as these are frequently used in professional soccer to manage travel-related sleep disturbances.

### Body composition

Assessed via tetrapolar electrical bioimpedance (Sanny, BIA1011AF) following standard prerequisites: 4-hour fast, 24-hour abstinence from alcohol and caffeine, and 12-hour rest from vigorous exercise [22]. Participants remained in dorsal decubitus for 10 min before electrode placement on the right hand and foot.

### Heart rate variability (HRV)

Recorded using a 10-lead Wincardio USB electrocardiograph (Micromed) with participants at rest in dorsal decubitus for 10 min. RR intervals were analyzed using Kubios HRV 2.0 (Biosignal Analysis and Medical Imaging Group, Kuopio, Finland). Time-domain variables included RR intervals (ms), RMSSD (ms), and SDNN (ms); frequency-domain variables included LF (ms²), HF (ms²), LF (nu), HF (nu), and LF/HF ratio, in accordance with Task Force standards.¹⁴ Blood pressure was measured post-recording using an automatic monitor (Omron HEM-7320). To minimize circadian influence, HRV was recorded in the morning (07:30–09:00 AM), following a minimum recovery window of 24 h after the last training session (predominantly low-intensity technical drills).

### Cardiorespiratory capacity

VO₂max was estimated using the Yo-Yo Intermittent Recovery Test Level 2 [23], selected for its high specificity for intermittent sport athletes [24, 25]. A familiarization session was performed 48 h before the official assessment to ensure protocol reliability. To ensure strict standardization, all assessments were conducted at the same time of day [between 08:00 and 10:00 AM], on the same [natural grass pitch], and under similar environmental conditions. Following a standardized [15-minute] warm-up consisting of [ light jogging and dynamic stretching], athletes received standardized verbal instructions. The test consisted of 20-meter shuttle runs at a progressively increasing pace dictated by an audio signal, interspersed with 10-second active recovery periods, until voluntary exhaustion or failure to maintain the required pace on two consecutive trials. Strong verbal encouragement was provided throughout to ensure maximal effort, and all tests were supervised by the same experienced assessors to eliminate inter-rater variability. Aerobic power was then calculated from the total distance covered using the equation:

$$VO_2max\;(ml.kg\:-\:1.min\:-\:1)\;=\;Total\;Dis\tan ce\;Covered\;(m)\;\times\;0.0084\:+\:36.4$$

### Statistical analysis

Data normality and variance homogeneity were verified using Shapiro-Wilk and Levene’s tests, respectively. Parametric data are expressed as mean ± SD; non-parametric data as median and interquartile range; categorical variables as absolute and relative frequencies. Group comparisons used one-way ANOVA with Tukey’s post hoc test (homogeneous variances) or Welch’s ANOVA with Games-Howell post hoc test (heterogeneous variances). Associations between categorical variables were assessed by Pearson’s Chi-Square or Fisher’s exact tests, with effect sizes interpreted via Cramér’s V (weak ≈ 0.10, moderate ≈ 0.30, strong ≈ 0.50). Relationships between physiological variables were assessed by simple linear regression and Pearson’s correlation, with magnitudes classified as small (0.10–0.29), medium (0.30–0.49), or large (> 0.50) [26]. All analyses were conducted in Jamovi (v. 2.6.4) and GraphPad Prism (v. 9.0), with significance set at *p* < 0.05.

## Results

Demographic and body composition characteristics are presented in Table 1. The professional group was older, taller, and showed higher body mass index and body fat percentage than the youth categories (all *p* ≤ 0.01). Body mass and muscle mass were not significantly different between groups, and playing position distribution was similar across categories (*p* = 0.73).

**Table 1:** Comparison of groups regarding body composition and field position among the SUB-15, SUB-17, and Professional (PRO) soccer categories

VARIABLES	AGE GROUPS				
	U-15 (*n* = 12)	U-17 (*n* = 15)	PRO (*n* = 26)	*p*-value	η^2^
Age	15,0(14,0–15,0^α^)	16,0 (16,0–17,0^α^)	22,0 (21,0–24,8^α^) ^#^	< 0,001*	0,74
Body mass (kg)	63,3 ± 10,8	64,87 ± 6,8	72,2 ± 9,36	0,08	0,17
Height (m)	1,68 ± 0,06	1,72 ± 0,06	1,76 ± 0,07 ^#^	0,01*	0,15
BMI (kg/m²)	22,2 ± 2,45	21,8 ± 2,02	23,3 ± 2,38 ^#^	0,01*	0,08
Body fat (%)	11,4 ± 3,52	11,7 ± 3,56	17,6 ± 5,40 ^#^	< 0,001*	0,31
Muscle mass (%)	46,1 ± 4,11	46,6 ± 2,29	48,9 ± 4,91	0,09	0,08
Playing position					
Goalkeeper	2 (16,7%)	2 (13,3%)	3 (11,5%)		
Center-back	3 (25%)	3 (20%)	2 (7,7%)		
Right-back	1 (8,3%)	1 (6,7%)	2 (7,7%)	0,73	
Left-back	2 (16,7%)	2 (13,3%)	2 (7,7%)		
Midfielder	1 (8,3%)	1 (6,7%)	9 (34,6%)		
Defensive midfielder	1 (8,3%)	2 13,3%)	2 (7,7%)		
Forward	2 (16,7%)	4 (26,7%)	6 (23,1%)		

Note: Data are mean ± SD or median (Q1–Q3). One-way ANOVA with Tukey post hoc test was used for continuous variables; chi-square test was used for categorical variables. # indicates a significant difference between the professional group and the youth categories in the post hoc comparison (*p* < 0.05). * indicates a significant overall group effect (*p* < 0.05). η² denotes eta squared

Physical performance, hemodynamic, autonomic, and sleep variables are detailed in Table 2. Professional athletes demonstrated significantly higher VO₂max (50.5 ± 4.56 vs. 40.1 ± 3.14 and 42.6 ± 5.70 ml·kg⁻¹·min⁻¹; *p* < 0.001; η² = 0.53) and greater distance covered (1979 ± 235 m; *p* < 0.001; η² = 0.55). Resting heart rate was significantly lower in professionals (54.9 ± 8.65 bpm; *p* = 0.03), reflecting greater cardiovascular adaptation. Blood pressure values were within normotensive ranges across all groups (SBP: *p* = 0.14; DBP: *p* = 0.26). Time-domain HRV analysis revealed significantly higher RMSSD in professionals (82.8 ± 17.5 ms) compared to U-15 (58.8 ± 8.61 ms) and U-17 (67.6 ± 10.1 ms; *p* < 0.001; η² = 0.35), alongside longer RR intervals (1079.8 ± 201.5 ms; *p* = 0.01). SDNN did not differ significantly (*p* = 0.47). In the frequency domain, LF (nu) was higher in professionals (50.0 ± 18.44; *p* = 0.01; η² = 0.16), while HF (nu) was lower (51.1 ± 17.48; *p* = 0.01), and the LF/HF ratio showed a trend toward sympathetic predominance in professionals (*p* = 0.06; η² = 0.05). Sleep efficiency was significantly greater in professionals (90.7 ± 6.13%; *p* = 0.01; η² = 0.23), as was the global PSQI score (4.84 ± 1.82; *p* = 0.02).

**Table 2:** Comparison of performance, hemodynamic, autonomic, and sleep quality component variables among the SUB-15, SUB-17, and professional soccer categories

VARIABLES	AGE GROUPS				
	SUB-15 (*n* = 12)	SUB-17 (*n* = 15)	PRO (*n* = 26)	*p*-value	η^2^
Physical performance					
VO₂max (mL·kg⁻¹·min⁻¹)	40,1 ± 3,14 ^**#**^	42,6 ± 5,70^**#**^	50,5 ± 4,56	< 0,001*	0,53
Distance covered (m)	1334 ± 298 ^#^	1496 ± 279 ^#^	1979 ± 235	< 0,001*	0,55
Hemodynamic variables					
HR (bpm)	63,9 ± 9,94 ^#^	61,4 ± 13,6	54,9 ± 8,65	0,03*	0,12
SBP (mmHg)	119,7 ± 7,71	116,6 ± 8,95	122,4 ± 9,45	0,14	0,07
DBP (mmHg)	66,2 ± 5,81	68,7 ± 6,91	68,7 ± 6,91	0,26	0,05
Autonomic variables					
Time domain					
RR (ms)	926,4 ± 201,3	886,7 ± 217,6 ^#^	1079,8 ± 201,5	0,01*	0,16
RMSSD (ms)	58,8 ± 8,61 ^#^	67,6 ± 10,1^#^	82,8 ± 17,5	< 0,001*	0,35
SDNN (ms)	61,9 ± 14,6	61,1 ± 13,5	55,7 ± 18,6	0,47	0,03
Frequency domain					
LF (ms^2^)	468 (0,31 − 0,99^α^)	744 (0,49 − 2,33^α^)	810 (0,58 − 1,41^α^)	0,33	0,02
HF (ms^2^)	1458 (0,91 − 2,10^α^)	936 (0,63 − 1,96^α^)	1234(0,55 − 1,66^α^)	0,48	0,02
LF (nu)	32,2 ± 15,35 ^#^	44,8 ± 11,30	50,0 ± 18,44	0,01*	0,16
HF (nu)	66,6 ± 15,23^#^	52,9 ± 8,67	51,1 ± 17,48	0,01*	0,15
LF/HF	0,32 (0,30 − 0,80^α^)	0,63 (0,52 − 0,98^α^)	0,74 (0,53 − 0,98^α^)	0,06	0,05
Sleep quality					
Global sleep quality	3,41 ± 1,64	3,00 ± 1,25	4,84 ± 1,82	0,02*	0,09
Sleep efficiency (%)	86,6 ± 8,25 ^#^	82,5 ± 12,2 ^#^	90,7 ± 6,13	0,01*	0,23

Note. Data are presented as mean ± standard deviation, or median (Q1–Q3) for non-normally distributed variables; n, sample size (relative frequency, %). Between-group comparisons were performed using one-way ANOVA followed by Tukey post hoc test for continuous variables, and chi-square test for categorical variables. VO₂max, maximal oxygen uptake; HR, heart rate; SBP, systolic blood pressure; DBP, diastolic blood pressure; RR, interval between consecutive heartbeats; RMSSD, root mean square of successive differences between normal RR intervals; SDNN, standard deviation of normal RR intervals; LF, low-frequency component of HRV spectrum; HF, high-frequency component of HRV spectrum; LF/HF, ratio between low- and high-frequency components; η², eta squared. * Statistically significant overall group effect (*p* ≤ 0.05); # Significant difference from the professional group in Tukey post hoc pairwise comparisons (*p* < 0.05)

PSQI domain analysis showed that sleep efficiency > 85% was most prevalent in professionals (84.6%; *p* = 0.001; X² = 0.40). Sleep medication use was significantly higher among professionals (53.8%; *p* = 0.001; X² = 0.43). However, exploratory sub-analyses within the professional group revealed that the inclusion of this variable did not significantly alter the primary outcomes, as no significant differences in RMSSD or VO₂max were observed between medication users and non-users (*p* > 0.05). Moderate daytime sleepiness was reported by 33.3% of U-15 athletes, compared to none in the professional group (*p* = 0.002; X² = 0.44). Global sleep quality was rated as “good” by the majority in all categories (*p* = 0.88) (Table 3).

**Table 3:** Analysis of sleep quality and its domains using the Pittsburgh Sleep Quality Index (PSQI) among UNDER-15, UNDER-17, and professional soccer categories

VARIABLES		AGE GROUPS			
Subjective quality	U-15 (*n* = 12)	U-17 (*n* = 15)	PRO (*n* = 26)	*p*-value	X^2^
Very good	4 (33,3%)	4 (26,7%)	9 (34,6%)		
Good	8 (66,7%)	11 (73,3%)	17 (65,4%)	0,86	0,07
Poor	0 (0,0%)	0 (0,0%)	0 (0,0%)		
Sleep latency (min)					
< 15	9 (75,0%)	10 (66,7%)	9 (34,6%)		
16–30	1 (8,3%)	3 (20,0%)	10 (38,5%)	0,11	0,26
31–60	2 (16,7%)	2 (13,3%)	7 (26,9%)		
Sleep duration (h)					
< 5	1 (8,3%)	0 (0,0%)	0 (0,0%)		
5–6	0 (0,0%)	0 (0,0%)	3 (11,5%)	0,15	0,25
> 7	11 (91,7%)	15 (100,0%)	23 (88,5%)		
Sleep efficiency (%)					
< 65	2 (16,7%) ^$^	2 (13,3%) ^$^	0 (0,0%)		
65–74	2 (16,7%) ^$^	3 (20,0%) ^$^	0 (0,0%)		
75–84	5 (41,6%) ^$^	4 (26,7%) ^$^	4 (15,4%)	*0,001	0,40
> 85	3 (25,0%)	6 (40,0%)	22 (84,6%) ^#^		
Sleep disturbances					
None	3 (25,0%)	2 (13,3%)	2 (7,7%)		
< 1	7 (58,4%)	7 (46,7%)	21 (80,8%)		
entre 1–2	1 (8,3%)	4 (26,7%)	3 (11,5%)	0,12	0,28
≥ 3	1 (8,3%)	2 (13,3%)	0 (0,0%)		
Use of sleep medication					
None	11 (91,7%) ^$^	14 (93,3%) ^$^	12 (46,2%)		
1	1 (8,3%) ^$^	0 (0,0%)	0 (0,0%)	*0,001	0,43
< 1	0 (0,0%)	1 (6,7%)	6 (23,1%) ^#^		
1–2	0 (0,0%)	0 (0,0%)	8 (30,7%) ^#^		
Daytime sleepiness					
None	5 (41,7%)	6 (40,0%)	17 (65,4%) ^#^		
Mild	3 (25,0%) ^$^	7 (46,7%) ^$^	9 (34,6%)		
Moderate	4 (33,3%) ^$^	0 (0,0%)	0 (0,0%)	*0,002	0,44
Severe	0 (0,0%)	2 (13,3%) ^$^	0 (0,0%)		
Global sleep quality					
Good	10 (83,3%)	13 (86,7%)	21 (80,8%)		
Poor	2 (16,7%)	2 (13,3%)	5 (19,2%)	0,88	0,06
Disorder	0 (0,0%)	0 (0,0%)	0 (0,0%)		

*PSQI* Pittsburgh Sleep Quality Index, absolute frequency (relative frequency %), n, sample size, *X*^2^ Chi-square test, *$* comparison of U-15 and U-17 categories with professionals (*p* ≤ 0.05); #, comparison of professionals with U-15 and U-17 categories (*p* ≤ 0.05); p, *Fisher’s Exact test;* V, *Cramer’s V and Phi tests*. Source: Created by the author (2025)

A significant association was found between RMSSD levels and global sleep quality (*p* = 0.004; X² = 0.50), with athletes in higher RMSSD ranges predominantly classified as good sleepers. Athletes with RMSSD 0–30 ms all presented poor sleep quality (Table 4).

**Table 4:** Association between RMSSD index and global sleep quality among U-15, U-17, and professional soccer players

	RMSSD CLASSIFICATION							
		> 100 (ms)		61–100(ms)	31–60 (ms)	0–30 (ms)	***p*** **-value**	**X** ^**2**^
GLOBAL SLEEP QUALITY	Good		3 (60,0%)	25 (86,2%)	16 (94,1%)	0 (0,0%)		
	Poor	2 (40,0%)		4 (13,8%)	1 (5,9%)	2 (100%)	0,004*	0,50^⁑^
	Sleep disturbance	0 (0,0%)		0 (0,0%)	0 (0,0%)	0 (0,0%)		
	Total	5		29	17	2	n (53)	100%

*PSQI* Pittsburgh Sleep Quality Index, *RMSSD* root mean square of successive differences between normal RR intervals, *ms* milliseconds squared; absolute frequency (relative frequency %), *n* sample size, *X*^2^ Chi-square test, ⁑ significant effect size, *pFisher’s Exact test,* V *Cramer’s V and Phi tests*. Source: Created by the author (2025)

RMSSD was significantly associated with VO₂max classification (*p* = 0.01; X² = 0.40), with athletes in the > 100 ms range predominantly showing “Excellent” (40%) or “Very good” (40%) aerobic capacity (Table 5).

**Table 5:** Association between RMSSD index and VO_2_max among U-15, U-17, and professional soccer players

	RMSSD CLASSIFICATION						
	VO₂max Classification	> 100 (ms)	61–100(ms)	31–60 (ms)	0–30 (ms)	*p*-value	X^2^
VO_2_ MAX	Excellent	2 (40,0%)	1 (3,4%)	0 (0,0%)	0 (0,0%)		
	Good	1 (20,0%)-	14 (48,3%)	3 (17,6%)	0 (0,0%)		
	Very good	2 (40,0%)	5 (17,2%)	2 (11,8%)	0 (0,0%)	0,01	0,40
	Average	0 (0,0%)	4 (13,8%)	6 (35,3%)	1 (50,0%)		
	Poor	0 (0,0%)	5 (17,2%)	6 (35,3%)	1 (50,0%)		
	Total	5	29	17	2	n (53)	100%

*RMSSD*, root mean square of successive differences between adjacent normal RR intervals; *VO*₂ max, maximum oxygen uptake; *ms*², meters per second squared; absolute frequency (relative frequency %); n, sample size; *X*²: Chi-squared test; *p, Fisher’s Exact test; V, Cramer’s V and Phi test; Source: Prepared by the author (2025)

Correlation analyses conducted separately by competitive category showed distinct patterns across groups. In the professional group, RMSSD was positively correlated with sleep efficiency (*r* = 0.569, *p* = 0.002) and VO₂max (*r* = 0.600, *p* = 0.001), whereas no significant association was observed with resting heart rate. In the SUB-15 category, RMSSD was negatively correlated with resting heart rate (*r* = -0.578, *p* = 0.049) and sleep efficiency (*r* = -0.658, *p* = 0.020), and no association was found with VO₂max. In the SUB-17 category, RMSSD was not significantly associated with resting heart rate, sleep efficiency, or VO₂max. These findings indicate that the associations observed in the pooled sample were not uniform across competitive categories (Table 6).

**Table 6:** Correlations between RMSSD, sleep efficiency, resting heart rate, and VO₂max by competitive category

Variable	SUB-15 *r* (*p*)	SUB-17 *r* (*p*)	PRO *r* (*p*)
RMSSD vs. resting heart rate	-0.578 (0.049)	0.124 (0.660)	-0.034 (0.868)
RMSSD vs. sleep efficiency	-0.658 (0.020)	-0.361 (0.187)	0.569 (0.002)
RMSSD vs. VO₂max	0.000 (1.000)	0.041 (0.883)	0.600 (0.001)

Values are Pearson’s correlation coefficients **(r)**, with **p** values in parentheses. RMSSD, root mean square of successive differences between adjacent normal RR intervals; VO₂max, maximal oxygen uptake. Results should be interpreted cautiously, as subgroup analyses are exploratory and may be influenced by age, maturation, training status, and sleep medication use

**Fig. 1:**
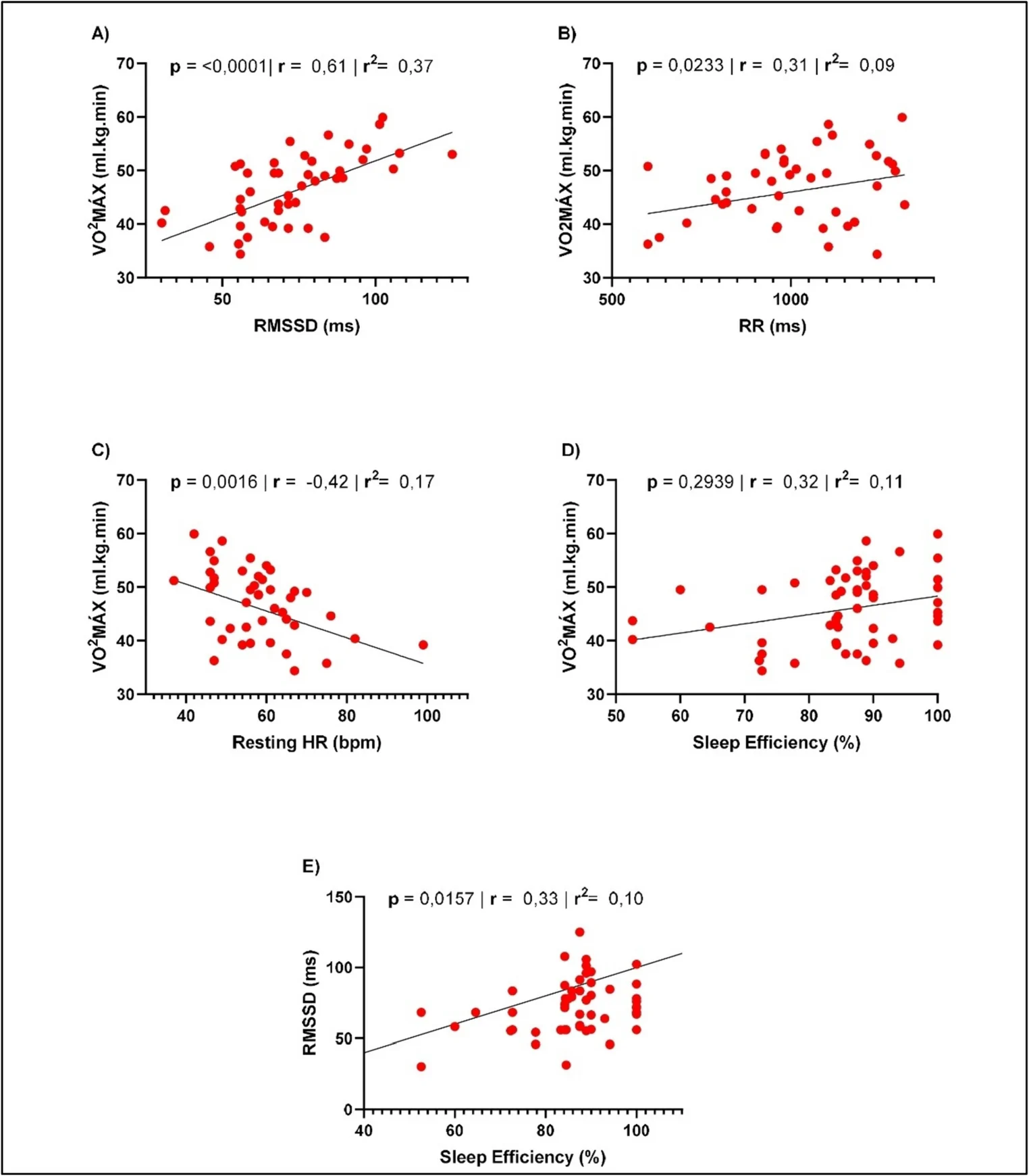
Correlation between maximum oxygen uptake (VO_2_ max), resting heart rate (HR), HRV variables (RMSSD and RR intervals), and sleep efficiency in soccer players from the UNDER-15, UNDER-17, and professional categories. **A**: Correlation VO^2^Max vs RMSSD; **B**: Correlation VO^2^Max vs RR; **C**: Correlation VO^2^Max vs HR; **D**: Correlation between VO₂max and sleep efficiency; **E**: Correlation between RMSSD and sleep efficiency; Pearson correlation test; VO^2^max, maximum oxygen uptake; RR, interval between consecutive heartbeats, expressed in milliseconds (ms); RMSSD, root mean square of successive differences between normal adjacent RR intervals within a time interval, expressed in ms; HR, Heart rate, expressed in bpm; Sleep efficiency, component of the Pittsburgh Sleep Quality Index (PSQI), expressed in %; r^2^, coefficient of determination; r, Pearson linear correlation; p, ≤ 0.05. Source: Prepared by the author

Pearson correlations revealed a moderate positive association between VO₂max and RMSSD (*r* = 0.61; *p* < 0.001; r² = 0.37), a positive but weaker correlation with RR intervals (*r* = 0.31; *p* = 0.023; r² = 0.09), and a significant negative correlation with resting heart rate (*r* = − 0.42; *p* = 0.002; r² = 0.17). A positive and significant correlation between RMSSD and sleep efficiency was also demonstrated, while the association between VO_2_max and sleep efficiency showed clinical relevance despite not reaching statistical significance (Fig. 1). 

## Discussion

This study investigated the association between sleep efficiency and cardiac autonomic modulation in young and professional soccer players during pre-season. The main findings confirm positive associations among RMSSD, global sleep quality, and VO₂max, as well as significant inter-category differences in autonomic regulation, aerobic performance, and specific sleep domains.

Regarding body composition, the results showed that professional athletes presented higher body fat percentage and higher muscle mass compared to youth players (Table 1). While some literature suggests that elite status is often associated with lower adiposity [27, 28], the higher body fat percentage observed in the professional group in this study compared to U-15 and U-17 categories may be attributed to their significantly greater total body mass and advanced biological maturation [29, 30]. Although relative muscle mass (%) did not differ significantly between groups in our sample, the overall body composition profile of the professionals is characterized by a significantly greater total body mass. We fully acknowledge that these morphological differences between adult professionals and youth categories (U-15 and U-17) are largely driven by advanced biological maturation. This maturational stage, acting synergistically with the cumulative effects of sports selection and chronic strength training typical of the professional environment, constitutes a key determinant of the superior physical performance observed in adult athletes [29, 30].

In addition to biological maturation, differences in the duration and structure of the pre-season period may also have contributed to the superior autonomic and physiological profile observed in the professional athletes. While the professional group followed a 4-week, more structured and periodized program, the youth categories had a shorter period of approximately 3 weeks, with less control and monitoring of the training load [31]. This scenario is consistent with evidence showing greater monotony and strain in less structured training programs for young athletes [31], in addition to reinforcing the previously demonstrated effects of pre-season training on physiological variables in young soccer players [12]. Thus, both the training structure and the maturational stage should be considered when interpreting the inter-category differences observed in this study.

The superior VO₂max of professional athletes reflects the cumulative effects of structured training on oxidative capacity and cardiovascular efficiency [32, 33]. However, as expected when comparing adult athletes to adolescents, this superior aerobic capacity is also strongly supported by the natural structural and functional maturation of the cardiopulmonary system that occurs with age. The significantly greater distance covered by the professional group supports the idea that long-term training promotes aerobic adaptations that optimize physical performance [34]. The lower resting heart rate in professionals reflects training-induced vagal tone increases and ventricular adaptations [35], consistent with the broader cardiovascular adaptation observed in this group. The absence of between-group differences in blood pressure is consistent with previous reports showing this variable to be less sensitive to training-related changes [36].

The significantly higher RMSSD and longer RR intervals observed in professionals are consistent with greater parasympathetic modulation, a marker commonly associated with chronic training adaptation [11]. However, it is essential to consider that cardiac autonomic modulation also undergoes significant changes during biological maturation. The transition from adolescence to adulthood is naturally accompanied by an increase in vagal tone and physiological reductions in resting heart rate [30]. Therefore, the observed autonomic differences between youth and professional players likely reflect a synergistic effect of normal maturational changes and chronic training adaptations. The positive correlation between RMSSD and sleep efficiency suggests that sleep quality may be associated with cardiac autonomic recovery, although the cross-sectional design and single-timepoint measurement of this study do not allow for causal or mechanistic conclusions regarding the role of sleep-mediated recovery in athletic performance [13]. Conversely, the lower vagal modulation observed among athletes with poorer sleep quality may indicate a tendency toward sympathetic predominance, a pattern that has been associated with training overload and performance decrements in previous studies [5]. The ergoreflex mechanism has been proposed as a potential contributor to these autonomic adaptations, as chronic optimization of metaboreflex and mechanoreflex pathways through training is thought to increase vagal tone and oxidative efficiency,⁴⁴ a hypothesis that appears consistent with the higher RMSSD, resting bradycardia, and longer RR intervals observed in professionals in the present study [37, 38].

Although global sleep quality was rated as good across all categories, relevant between-group differences emerged in sleep efficiency, latency, and medication use. Professional athletes showed higher sleep efficiency but longer latency, possibly reflecting competitive stress or scheduling constraints [39]. The significantly higher prevalence of sleep medication use among professionals (53.8%) is noteworthy [40], underscoring the need for non-pharmacological sleep management strategies in this population. Daytime sleepiness in approximately one-third of U-15 athletes, despite overall good subjective quality, may reflect the influence of academic demands, training load, and screen exposure [41, 42].

### Limitations

This study presents some limitations. Its cross-sectional design precludes inferences regarding causality. Importantly, this design also makes it challenging to fully isolate the specific autonomic adaptations induced by sports training from the normal physiological changes associated with biological maturation. Because pubertal advancement naturally increases parasympathetic modulation, the observed autonomic differences likely reflect a complex interaction between normal maturational development and specific training demands. Furthermore, sleep quality was assessed using the PSQI rather than objective methods such as actigraphy or polysomnography. Although the use of sleep medication was recorded, it was not controlled for in the primary analyses, representing a potential confounding factor. Data were collected at a single time point during the second week of the preseason, limiting the interpretation of temporal changes in sleep and autonomic responses. Additionally, the relatively small subgroup sample sizes warrant a cautious interpretation of the comparisons, which is why effect sizes were emphasized to evaluate the magnitude of differences independent of sample size constraints. Finally, the study was conducted with athletes from a single club and did not strictly control for external variables such as diet and hydration status, which may limit the generalizability of the findings. Future longitudinal studies should include repeated assessments of sleep, HRV, maturational status, and training load throughout the entire preseason.

## Conclusion

Professional soccer players demonstrated superior physiological adaptations, higher VO₂max, resting bradycardia, and greater vagal autonomic modulation compared to U-15 and U-17 athletes during pre-season. Sleep efficiency correlated positively with RMSSD and showed clinical relevance to VO₂max, highlighting sleep as a key modulator of cardiac autonomic recovery and aerobic performance. Despite greater sleep efficiency, professionals exhibited longer sleep latency and higher medication use, indicating that sleep management remains a critical area of intervention across all competitive categories. These findings support the use of HRV and PSQI as complementary monitoring tools in the integrated assessment of soccer players across competitive levels.

## Data Availability

The data supporting this study are available from the corresponding author upon reasonable request.
